# Ultrastructural changes of erythrocytes in whole blood after exposure to prospective *in silico*-designed anticancer agents: a qualitative case study

**DOI:** 10.1186/0717-6287-47-39

**Published:** 2014-09-04

**Authors:** Lisa Repsold, Thandi Mqoco, Elize Wolmarans, Sandra Nkandeu, Joji Theron, Tomek Piorkowski, Peet du Toit, Dirk van Papendorp, Annie Margaretha Joubert

**Affiliations:** Department of Physiology, School of Medicine, Faculty of Health Sciences, University of Pretoria, Pretoria, South Africa

**Keywords:** Whole blood, Morphology, ESE-15-ol, ESE-16, Anticancer, Erythrocytes

## Abstract

**Background:**

Novel, *in silico*-designed anticancer compounds were synthesized in our laboratory namely, 2-ethyl-3-*O*-sulphamoyl-estra-1,3,5(10),15-tetraen-17-ol (ESE-15-ol) and 2-ethyl-3-*O*-sulphamoyl-estra-1,3,5(10)16-tetraene (ESE-16). These compounds were designed to have improved bioavailability when compared to their source compound, 2-methoxyestradiol. This theoretically would be due to their increased binding affinity to carbonic anhydrase II, present in erythrocytes. Since the novel compounds under investigation are proposed to be transported within erythrocytes bound to carbonic anhydrase II, the morphological effect which they may exert on whole blood and erythrocytes is of great significance. A secondary outcome included revision of previously reported procedures for the handling of the whole blood sample.

The purpose of this study was twofold. Firstly, the ultrastructural morphology of a healthy female’s erythrocytes was examined via scanning electron microscopy (SEM) after exposure to the newly *in silico*-designed compounds. Morphology of erythrocytes following exposure to ESE-15-ol and ESE-16 for 3 minutes and 24 hours at 22°C were described with the use of SEM. The haemolytic activity of the compounds after 24 hours exposure were also determined with the ex vivo haemolysis assay. Secondly, storage conditions of the whole blood sample were investigated by determining morphological changes after a 24 hour storage period at 22°C and 37°C.

**Results:**

No significant morphological changes were observed in the erythrocyte morphology after exposure to the novel anticancer compounds. Storage of the whole blood samples at 37°C for 24 hours resulted in visible morphological stress in the erythrocytes. Erythrocytes incubated at 22°C for 24 hours showed no structural deformity or distress.

**Conclusions:**

From this research the optimal temperature for *ex vivo* exposure of whole blood samples to ESE-15-ol and ESE-16 for 24 hours was determined to be 22°C. Data from this study revealed the potential of these compounds to be applied to *ex vivo* study techniques, since no damage occurred to erythrocytes ultrastructure under these conditions. As no structural changes were observed in erythrocytes exposed to ESE-15-ol and ESE-16, further *ex vivo* experiments will be conducted into the potential effects of these compounds on whole blood. Optimal incubation conditions up to 24 hours for whole blood were established as a secondary outcome.

## Background

In our laboratory novel, *in silico*-designed anticancer compounds namely, 2-ethyl-3-*O*-sulphamoyl-estra-1,3,5(10),15-tetraen-17-ol (ESE-15-ol) and 2-ethyl-3-*O*-sulphamoyl-estra-1,3,5(10)16-tetraene (ESE-16) were recently synthesized [1]. The aim of the compounds’ design was not only to increase potency, but also to improve their bioavailability when compared to their source compound, 2-methoxyestradiol (2ME) [1]. Improved bioavailability was achieved with an addition of a sulphamoylated group to the compound which increases their binding affinity to carbonic anhydrase II (CAII) present in erythrocytes, thus bypassing the first pass of liver metabolism [1–3]. Increased anticancer potency was achieved with modifications at positions 3 and -17 of the compounds [4–9].

The antiproliferative influence of these compounds has been demonstrated on various cancer cell lines, particularly various breast and cervical cancer cell lines [1–3]. They were shown to suppress microtubule dynamics, causing mitotic spindle disruption, inhibition of cancer cell proliferation and subsequently induce cell death *in vitro* [1–3, 10, 11]. Additionally, the compounds inhibit the activity of carbonic anhydrase IX (CAIX), an enzyme that is over expressed in a variety of tumours and is known to promote acidification of the tumour microenvironment [12, 13]. The acidic environment, in turn, plays a role in promoting actions of growth factors and proteases involved in tumor progression [1]. *In vitro* results led to the *ex vivo* investigation of the influence of these two novel compounds on the morphology of platelets and whole blood*.*

In order to investigate the above-mentioned, previously reported literature procedures for storage of whole blood (WB) were revisited. WB is a controversial subject since temperature and length of storage impact on diagnostic markers including morphology, enzyme activity, haemolysis, membrane loss and platelet activation when determining possible pathophysiology [14–16]. Some researchers believe that WB can only be stored at room temperature for a maximum of 8 hours due to concerns over bacterial contamination, loss of 2,3-diphosphoglycerate (2,3-DPG) in erythrocytes, as well as preservation of Factor (F) VIII content in plasma [16]. It is recommended to cool blood to 4°C if it must be stored longer than 8 hours before processing or separation into separate components [16]. This, however, renders blood samples unsuitable for platelet preparation when determining cell numbers and pathological conditions [16].

Van der Meer *et al*. reported that WB can be stored at room temperature for up to 24 hours and this may reduce the risk of bacterial contamination and improve platelet yield and quality [16]. In addition, Benson & Skaar reported that WB stored at room temperature showed no signs of erythrocyte lysis nor significant alteration of plasma microDNA [14].

Erythrocytes are regarded as a reliable model for the study of oxidative stress [17, 18]. Their morphological changes can be recorded as indicators of oxidative stress, since erythrocytes are exposed to various adverse conditions including gas exchange where cells are exposed to hydroxyl radicals and inflammatory processes they are highly susceptible to oxidative stress [17, 18]. Storage of WB may affect erythrocyte morphology, since pH, gasses, bicarbonate and 2,3-DPG concentrations may be altered [15, 16].

The purpose of this study was to examine the external morphology of a healthy female’s erythrocytes using scanning electron microscopy (SEM) and the *ex vivo* haemolysis assay after exposure to newly *in silico*-designed potential anticancer drugs. A secondary outcome of the study was to determine the optimal incubation conditions for whole blood.

## Results and discussion

### Optimal temperature determination

**Figure 1:**
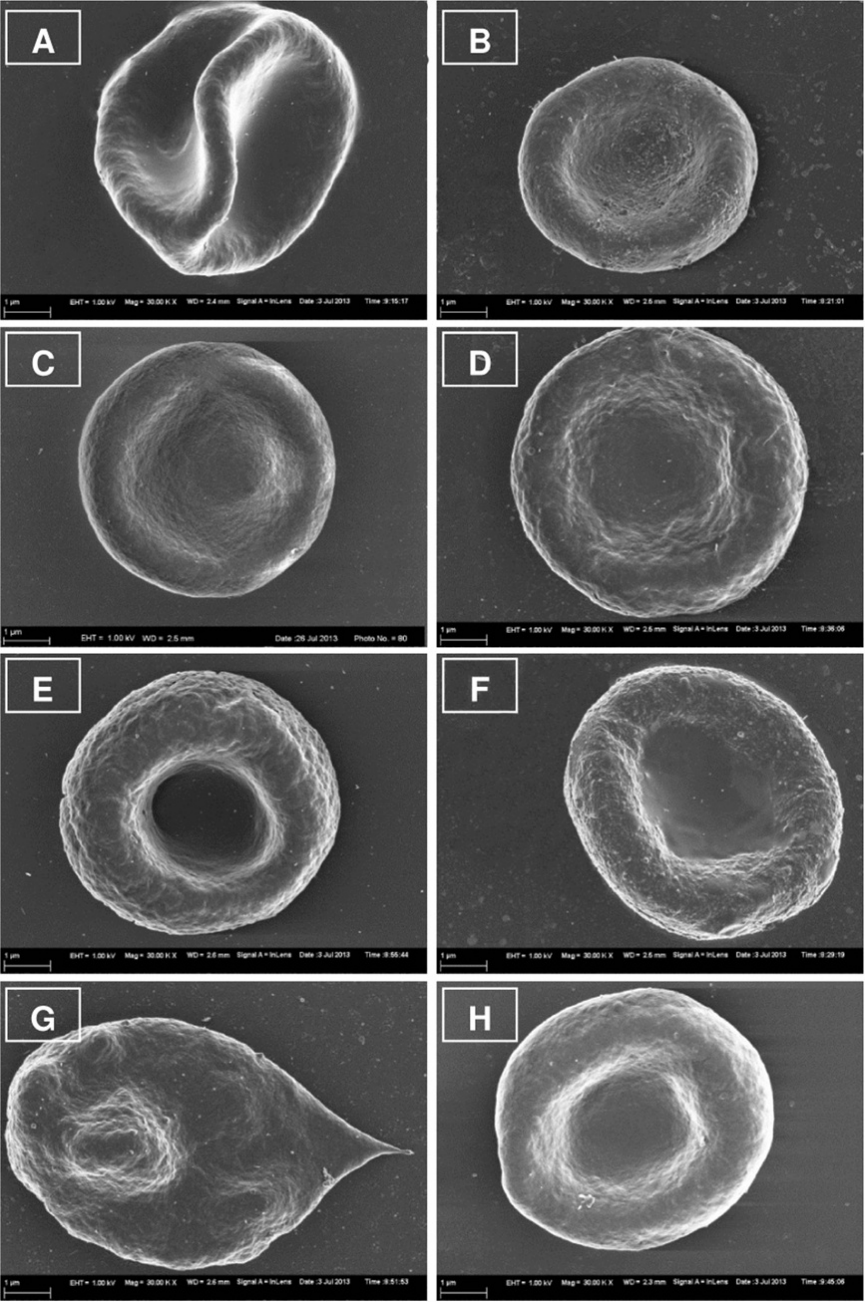
**SEM images of red blood cells after exposure at 37°C and 22°C for 22 hours.** Untreated control erythrocytes at 37°C **(A)** and 22°C **(B)**. Vehicle control erythrocytes at 37°C **(C)** and 22°C **(D)**. ESE-15-ol-treated erythrocytes at 37°C **(E)** and 22°C **(F)**. ESE-16-treated erythrocytes at 37°C **(G)** and 22°C **(H)**.

Whole blood was stored at 22°C and 37°C respectively to determine the optimal temperature at which erythrocytes retain normal morphology. Erythrocytes show normal morphology at 22°C when compared to 37°C, thus 22°C was selected for subsequent experiments. The morphology of erythrocytes (Figure 1A and C) at 37°C was clearly affected, indicating signs of stress including formation of extended projections, alteration of surface membrane structure to more bulbous structure and lengthening of erythrocytes. Erythrocytes exposed at 22°C (Figure 1B and D) showed normal biconcave structure of erythrocytes with no lengthening or blebbing.

### Immediate cytotoxicity

**Figure 2:**
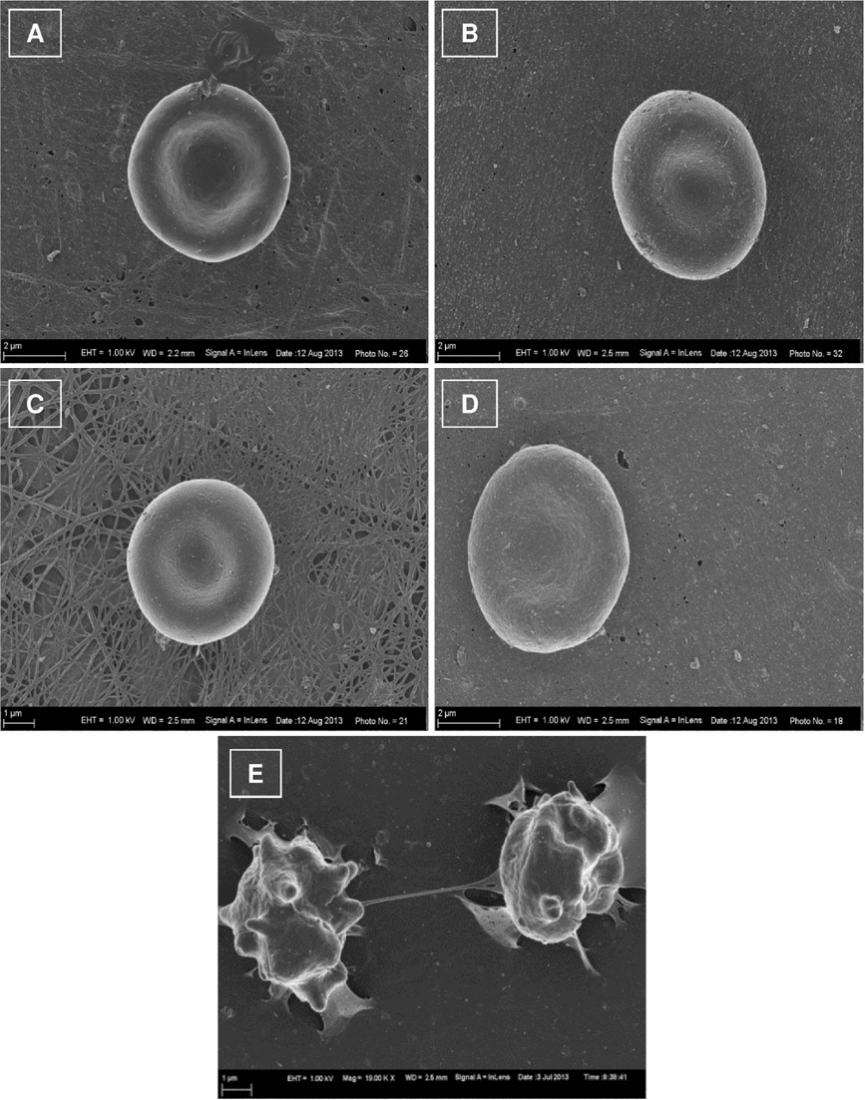
**SEM images of erythrocytes after 3 min exposure at 22°C. (A)** erythrocytes of untreated WB sample, **(B)** erythrocytes of WB sample treated with DMSO as vehicle control, **(C)** erythrocytes of WB sample treated with ESE-15-ol and **(D)** erythrocytes of WB sample treated with ESE-16 and **(E)** erythrocytes of WB sample treated with 1% DMSO (v/v) as positive control. No significant morphological changes in the erythrocytes treated with the compounds and controls were observed after 3 min.

Figure 2 indicates SEM images of erythrocytes after being exposed to ESE-15-ol and ESE-16 and the relevant control for 3 min (Figure 2C and D) and 24 hours (Figure 2E and F) at 22°C. Results revealed no significant morphological changes in erythrocytes after 3 min and 24 hours in WB samples exposed to the compounds and the control respectively. This indicates that the compounds do not cause an immediate or delayed stress reaction visible in the erythrocyte membrane of a healthy individual.

### Ex vivo erythrocyte haemolysis assay

**Figure 3:**
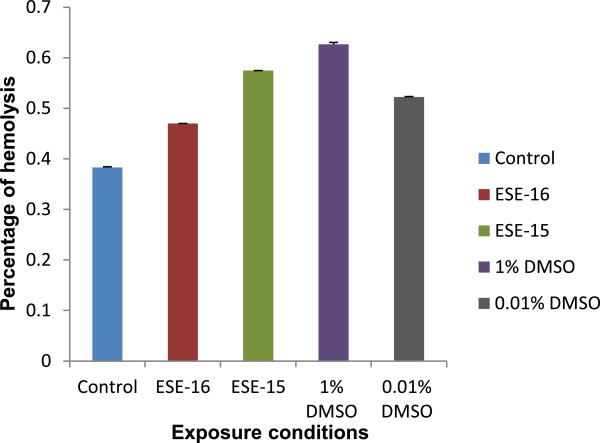
***Ex vivo*** **haemolysis assay.** The haemolytic activity of ESE-15-ol- and ESE-16-treated erythrocytes was found to be less than 1% as observed in the control samples. Standard deviation was indicated on the graph for 3 technical repeats.

The % of haemolysis for all control and exposed samples is represented in Figure 3. The results indicate that all samples exhibit less than 1% haemolytic activity. This data corresponds with SEM results showing that these novel compounds do not affect the morphology of the erythrocytes and have no cytotoxic effects.

This study investigated the possible immediate cytotoxicity which occurs within 3 min of exposure to the unique ESE-15-ol and ESE-16 anticancer compounds, which was compared to the morphological effects of WB exposure for 24 hours at room temperature (22°C). Results revealed that there was no significant damage to the structure of erythrocyte membranes when exposed to ESE-15-ol and ESE-16. This was also seen in the *ex vivo* haemolysis assay results which indicated the compounds had no significant haemolytic activity after 24 hours exposure. DMSO-treated erythrocytes at a concentration of 1% showed morphological alteration as expected for a positive control. However there was no statistically significant increase in haemolytic activity indicating that this concentration of DMSO does not rupture erythrocyte membranes.

In addition, WB stored at room temperature (22°C) showed significantly less morphological changes when compared to samples stored at 37°C. This is similar to the findings of Van der Meer *et al.* (2011) who demonstrated that the quality of WB stored at room temperature overnight was comparable to that of freshly processed WB [16]. In addition, Baumgarten *et al*. showed a decrease in platelet counts, as well as platelet aggregation when blood was stored at 4°C since platelets aggregate at low temperatures which results in the disintegration of the platelets [19]. It has also been suggested that storage of blood at room temperature provides more suitable haemostatic function for *ex vivo* experiments, since preservation of homeostasis and platelet function is optimal at room temperature [19].

Stander suggested that ESE-15-ol and ESE-16 reversibly bind to carbonic anhydrase II (CAII) in red blood cells in order to bypass the first pass metabolism in the liver due to release into the blood stream from CAII, resulting in increased bioavailability [1]. Since these compounds are hypothesised to be carried in the blood bound to CAII, the effect of these compounds on the morphology of erythrocytes is significant and results indicate the normal morphology of erythrocytes when exposed to these compounds which suggest future *in vivo* studies to be viable.

## Conclusions

This case study reports on the influence of ESE-15-ol and ESE-16 on the morphology of erythrocytes *ex vivo*. Results indicate that these compounds do not cause morphological damage to erythrocytes when exposed to whole blood for 3 min or 24 hours at room temperature (22°C). *Ex vivo* research may be superior when conducted at room temperature (22°C), since samples exposed at these temperatures showed less change in morphological structure when compared to samples incubated at 37°C. Data from this study reveal the potential of these compounds to be applied to *ex vivo* study techniques at 22°C. Future *ex vivo* investigations will focus on the other components within whole blood when exposed to ESE-15-ol and ESE-16.

## Methods

### Materials

Ethylene-diamine-tetra-acetic acid (EDTA) tubes and needles were acquired from Transpharm (Gauteng, SA). Phosphate-buffered saline (PBS) was purchased from Gibco-BRL (Invitrogen, Carlsbad, CA, USA). Dimethyl sulphoxide (DMSO) was supplied by Sigma-Aldrich Co (St. Louis, USA).

### Preparation of compounds

Since ESE-15-ol and ESE-16 were *in silico*-designed at the Bioinformatics and Computational Biology Unit, Department of Biochemistry, University of Pretoria, South Africa and are not commercially available. Synthesis of ESE-15-ol and ESE-16 was done by Ithemba Pharmaceuticals (Pty) Ltd (Modderfontein, Midrand, South Africa). ESE-15-ol and ESE-16 were dissolved in DMSO and the final concentration did not exceed 0.01% (v/v) in subsequent experiments [1]. Blood samples were exposed to 0.18 μM of ESE-15-ol and ESE-16 [1]. This concentration was selected since our laboratory established previously that ESE-15-ol and ESE-16 inhibited cell growth by 50% (GI_50_) at this concentration after 24 hours at 37°C *in vitro* [1]. Control samples included DMSO at a concentration of 0.01% as vehicle control, DMSO at a concentration of 1% (v/v) as a positive control and untreated WB as negative control [20].

### Method

#### Study design and sampling method

To conduct the case study, blood was collected from a healthy female individual aged 45 years who does not smoke or does not use any medication [21]. As breast cancer is the second leading cause of mortality of females globally and first leading cause in sub-Saharan Africa with poor survival rates, a female participant was chosen for this study, to determine possible future applications [22–24]. Even though the compounds tested in this study are derived from 17β-estradiol, the compounds do not bind selectively to estrogen receptors, yet rather to CAII and any hormonal fluctuations in a female participant would not have an effect on the drugs activity or binding [1–3].

The participant met the following exclusion criteria: chronic or acute illnesses, autoimmune diseases, hereditary diseases, hypertension, contraceptives, or smoking. Informed consent was obtained from the individual before collection of blood in EDTA tubes and blood samples were collected after an 8 hour period of fasting between 08:00-09:00 am. Platelet-rich plasma (PRP) was obtained by centrifuging the blood at 1000 rpm for 2 min and collecting plasma from the separated blood [25].

A time- and temperature-based study was conducted with the use of SEM to assess morphological changes on WB by exposing WB to ESE-15-ol and ESE-16 at time intervals of 3 min to determine possible immediate toxicity and 24 hours to determine effects of the compounds at this time interval. The latter is equivalent to the exposure time used for cancer cell lines in our laboratory [1–3]. WB samples were exposed for 24 hours at 22°C and 37°C respectively to determine optimal temperature at which the membrane morphology of erythrocytes remained normal and at which future *ex vivo* studies exposure to ESE-15-ol and ESE-16 may occur.

#### Scanning electron microscopy

Morphology of erythrocytes within WB samples exposed to ESE-15-ol and ESE-16 at time intervals of 3 min (to determine possible immediate toxicity) and 24 hours (to determine effects at this time interval equivalent to exposure of cancer cell lines) was determined via SEM. Morphology of WB samples when exposed to ESE-15-ol and ESE-16 for 24 hours at 22°C and 37°C respectively was also studied. Samples were viewed with the Zeiss ULTRA plus FEG-SEM at the Microscopy and Microanalysis Unit of the University of Pretoria, Pretoria, South Africa.

*Ex vivo* samples were prepared on glass plates with 10 μl (10^7^ platelets/ml) WB as a control, 10 μl WB exposed to ESE-15-ol and 10 μl WB exposed to ESE-16 respectively [25]. Glass plates with *ex vivo* samples were placed in 6 well plates and left to dry slightly, after which the samples were washed for 20 min in a 50% PBS: 50% distilled H_2_O solution. Samples were fixed with gluteraldehyde and PBS for 30 min and washed 3 times in PBS for 3 min each for subsequent secondary fixation in osmium for 15 min. Samples were washed 3 times each for 3 min and dehydrated for 3 min each in increasing concentration of ethanol, 30%, 50%, 70%, 90% and three times in 100% ethanol [25]. Samples were critically dried, mounted and carbon-coated and were viewed using the Zeiss ULTRA plus FEG-SEM (Carl Zeiss (Pty) Ltd, Johannesburg, South Africa). Qualitative SEM images were obtained from 3 independent experiments repeated for the participant. Representative images were included to represent all 3 repeats.

#### Ex vivo erythrocyte haemolysis assay

To quantitatively assess the membrane disrupting potential of ESE-15-ol and ESE-16 on erythrocytes, an *ex vivo* erythrocyte haemolysis assay was performed.

When the erythrocyte membrane is destroyed, haemoglobin is released [26]. The haemoglobin can then be isolated from the erythrocytes and spectrophotometrically quantified at an absorbance of 540 nm [26, 27].

Whole blood samples were placed in 96 well plates (200 μl of blood sample per well) and exposed to the compounds or the various controls as discussed above. The samples were then incubated at 22°C for 24 hours. After the incubation period, 20% Triton X-100 was added to designated samples as a positive control for erythrocytes heamolysis and incubated at 22°C for 10 min. Samples (200 μl) were then transferred to eppies and diluted ten times with PBS (pH 7.4). Samples were spun down at 10 000 × g for 10 min. Supernatant (100 μl) was transferred to a clean 96-well plate and absorbance determined at a wavelength of 540 nm with the use of an EL_x_800 Universal Microplate Reader (Bio-Tek Instruments Inc. Vermont, USA). For data analysis the background absorbance value was subtracted from all sample values and the average absorbance of the positive control samples were used to normalize all experimental data points to this mean absorbance value, representing 100% hemolysis. To calculate % hemolysis in each well relative to positive control, their values were multiplied by 100% [26, 27]. Results were obtained from 3 technical repeats for the participant.

## Ethics

Ethical clearance for the collection of blood was obtained from The Research Ethics Committee, Faculty Health Sciences, University of Pretoria which complies with ICH-GC.P guidelines (Ethics clearance number: 289/2013).
